# Disclosure of HIV status and adherence to antiretroviral treatment in children and adolescents from Lomé and Abidjan

**DOI:** 10.11604/pamj.2023.45.13.26795

**Published:** 2023-05-04

**Authors:** Tchaa Abalo Bakai, Jean Iwaz, Elom Ounoo Takassi, Anne Thomas, Tanoh Kassi François Eboua, Nagham Khanafer, Tchasso Kenao, Kariyiare Benjamin Goilibe, Esseboe Sewu, Nicolas Voirin

**Affiliations:** 1 Centre Africain de Recherche en Épidémiologie et en Santé Publique (CARESP), Lomé, Togo,; 2 Université de Lyon, Lyon, France,; 3 Université Lyon 1, Villeurbanne, France,; 4 Hospices Civils de Lyon, Pôle Santé Publique, Service de Biostatistique-Bioinformatique, Lyon, France,; 5 CNRS UMR 5558, Laboratoire de Biométrie et Biologie Évolutive, Équipe Biostatistique-Santé, Villeurbanne, France,; 6 Epidemiology and Modelling (EPIMOD), Dompierre-sur-Veyle, France,; 7 Centre Hospitalier Universitaire Sylvanus Olympio, Service de Pédiatrie, Lomé, Togo,; 8 Centre de Traitement Ambulatoire Pédiatrique (CTAP), Centre Hospitalier Universitaire de Yopougon, Abidjan, Côte d´Ivoire,; 9 Hospices Civils de Lyon, Hôpital Édouard Herriot, Service d´Hygiène, Épidémiologie et Prévention, Lyon, France

**Keywords:** Children, adolescents, aids, truth disclosure, patient compliance, university hospitals, Togo, Ivory Coast

## Abstract

**Introduction:**

in Africa, the proportion of minors with AIDS is ever increasing and adherence to treatment protocols is still suboptimal. The study investigated the conditions of HIV status disclosure and adherence to treatment in patients < 19 in two West African cities.

**Methods:**

in 2016, thirteen health professionals and four parents filled out questionnaires to identify problems and solutions relative to disclosure of HIV status and adherence to treatment in 208 children and adolescents seen at University Hospitals in Abidjan (Ivory Coast) and Lomé (Togo).

**Results:**

medians (extrema) of patients´ ages at start and end of status disclosure process were 10 (8-13) and 15 (13-17.5) years. In 61% of cases, disclosure was made individually after preparation sessions. The main difficulties were: parents´ disapproval, skipped visits, and rarity of psychologists. The solutions proposed were: recruiting more full-time psychologists, improving personnel training, and promoting patients´ “clubs”. One out of three respondents was not satisfied with patients´ adherence to treatments. The major reasons were: intake frequencies, frequent omissions, school constraints, adverse effects, and lack of perceived effect. Nevertheless, 94% of the respondents confirmed the existence of support groups, interviews with psychologists, and home visits. To improve adherence, the respondents proposed increasing the number of support groups, sustaining reminder phone calls and home visits, and supporting therapeutic mentoring.

**Conclusion:**

despite persisting disclosure and adherence problems, appropriate measures already put into practice still need to be taken further, especially through engaging psychologists, training counsellors, and promoting therapeutic support groups.

## Introduction

The proportion of adolescents among people living with HIV is ever increasing [1] and adolescents aged 10 to 19 years are among the most neglected categories in the global response to HIV, whereas AIDS is the second cause of death among adolescents worldwide and the first cause of death among adolescents in sub-Saharan Africa [2]. According to the last UNAIDS data 2017, nearly 67 000 children aged 0 to 9 years and 69 000 adolescents aged 10 to 19 years were newly infected during year 2017 [1]. In West Africa, in 2017, nearly 800 000 children and adolescents (0 to 19 years) were living with HIV, which is the third highest number worldwide after Eastern and Southern Africa.

During the conference on HIV/AIDS held in Dakar (16-18 January, 2019) and attended by Ministries of Health, HIV experts, and representatives of the United Nations, the African Union, the Economic Community of West African States, the Economic Community of Central African States, and the civil society, Marie-Pierre Poirier, the UNICEF Regional Director for West and Central Africa said: “The majority of children living with HIV in this region are not receiving care and treatment because they do not know they have HIV as they have not been tested.” Michel Sidibé, the Executive Director of UNAIDS, said: “Underlying issues including a lack of domestic investment, fragile health systems, user fees, gender inequality and widespread stigma and discrimination must urgently be addressed to remove barriers and save lives.” [1].

Despite advances in antiretroviral therapy (ART) coverage among Western and Central African HIV-infected children (26% in 2017 vs. 18% in 2014), this region still has the lowest ART coverage rate globally. In 2017, nearly 52 000 children and adolescents aged 0 to 19 years died from diseases linked to AIDS, of whom 34 000 deaths before the age of 5 years [1]. In West Africa, Togo and Ivory Coast are limited-resource countries where the management of pediatric HIV infections is a real challenge. Beside difficulties related to the disclosure of the HIV status to children or adolescents, there are problems of adherence to the ART and problems of transition of adolescents from pediatric to adult health services. To these are added complications linked to poverty, death of a parent, uncertainties regarding guardianship, isolation, and stigma that increase the likelihood of behavioral disorders, especially in adolescence [3-5].

In 2014, Togo and Ivory Coast agreed to participate in a project that involved designing, monitoring, and conducting a prospective multicentric project, the West Africa International epidemiologic Database to Evaluate AIDS (IeDEA). The project included the collection of data on children and adolescents living with HIV and followed-up within a well-defined cohort [5]. Five years after the launching of IeDEA project, the objective of the present study was to collect information on HIV status disclosure and adherence to ART in children and adolescents living with HIV in Lomé and Abidjan.

## Methods

### Study setting

Project COHADO, a subproject of IeDEA West Africa [6], was established in 2014 and 2017 in Togo and Ivory Coast only to collect information on adolescent problems linked with HIV infection and management in West Africa. This qualitative prospective multicentric study targeted a part of cohort COHADO. It was carried out in 2016 at two general pediatrics departments; precisely, from March 7^th^ to April 7^th^ at the *Centre de Traitement Ambulatoire Pédiatrique de Centre Hospitalier Universitaire de Yopougon* (Abidjan, Ivory Coast) and from April 8^th^ to May 10^th^ at the *Service de Pédiatrie* of *Centre Hospitalier Universitaire* Sylvanus Olympio (Lomé, Togo).

**Study participants:** the study consisted in a questionnaire given to: i) Six physicians or pharmacists working full-time at the university hospitals (3 at Yopougon and 3 at Lomé); ii) Two full- or part-time psychologists (1 at Yopougon and 1 at Lomé); iii) Five full-time mediators (3 at Yopougon and 2 at Lomé); and, iv) Four mothers of four adolescents living with HIV, two seen at Yopougon and two at Lomé.

### Design and conduction of the interviews

A focus group and a pre-test were carried out before questionnaire administration to check the questionnaire quality and amend and tune it as necessary. The 13 health professionals responded anonymously to the self-administered questionnaire. The four mothers answered the questionnaire during standardized semi-directed interviews conducted by a physician-epidemiologist and held at a university hospital during a visit for a child. The duration of the latter interviews was 30 to 45 minutes and used either French or a local dialect. As the follow-ups of the children included parents´ participation to awareness-raising and therapeutic education sessions, the interviewed mothers were fully able to understand the questions and give unambiguous answers. The agreement to the anonymous collection and analysis of the data was given by the Local Ethics Committee for Health Research of each hospital. All respondents gave their informed consent prior to participation in the interviews.

**Data analysis:** the anonymized answers of the 13 health professionals and the 4 mothers were collected, stored, and analyzed with Epi-info, version 7. Categorical data were summarized by counts and percentages and represented by histogram bars. Respondent verbatims underwent a thematic analysis of content.

## Results

Thirteen health professionals (7 at Abidjan and 6 at Lomé) and four mothers of adolescent patients (2 at Abidjan and 2 at Lomé) filled out fully the study questionnaire about 101 patients at Lomé and 107 patients at Abidjan.

### Disclosure of the HIV status

**Figure 1:**
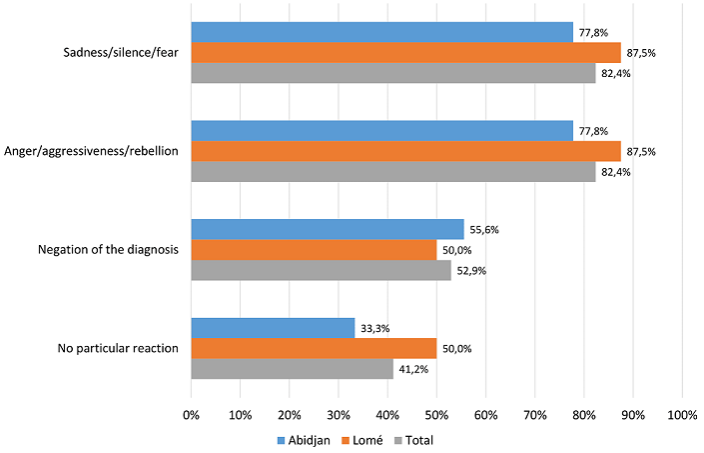
immediate reactions of children and adolescents at disclosure of their HIV status

Eleven respondents (64.7%) participated to the disclosure of the HIV status either through individual psychological preparation or via group counselling in the presence of parents or legal guardians. The median ages of the patients at start and end of the status disclosure process were 10 years (range: 8-13) and 15 years (range: 13-17.5). In 58.8% of cases, the disclosure was made on a case-by-case basis after several counselling sessions divided into three stages (pre-disclosure, disclosure, and post-disclosure). In HIV-infected children and adolescents, the most common immediate reactions to HIV status disclosure were anger, aggressiveness, revolt, sadness, prostration, and cries (Figure 1). The main difficulties in the disclosure process were: parents´ disapproval, failure to attend the scheduled visits, and unavailability of pediatric clinical psychologist (Table 1). The solutions proposed by the respondents to improve the disclosure process were: recruiting more full-time child and adolescent psychologists, improving the training of disclosure-involved personnel, establishing local “clubs” for this personnel, and promote parents´ awareness of the need to make the disclosures within the recommended time frame (Table 1).

**Table 1:** adherence to ART and disclosure of HIV status process (obstacles and solutions)

Questionnaire item	Number	Percentage
**Adherence to antiretroviral treatment**		
**Are there in your department support activities to improve adherence?**		
Yes	16	94.12
No	0	0
Missing answer	1	5.88
Total	17	100
**If yes, which activities?**		
Speech group	17	100
Adherence group	17	100
Individual consultation with a psychologist	16	94.12
Home visits	15	88.23
Therapeutic education	14	82.35
**What measures would you suggest to improve adherence?**		
Increase the number of speech and adherence club sessions	17	100
Maintain phone recalls and therapeutic education activities	16	94.12
Increase the number of home visits	16	94.12
Support therapeutic mentoring	14	82.35
Make injectable antiretrovirals available	7	41.18
**Disclosure of HIV status**		
**Do you regularly disclose HIV status?**		
Yes	11	64.71
No	6	35.29
Age at start of disclosure process (years)	10 [8-13]	
Age at end of disclosure process (years)	15 [13-17.5]	
**How the disclosure is usually made?**		
Individually, in 3 phases (Pre-disclosure, Disclosure, Post-disclosure)	10	58.82
Counseling, with parents agreement, psychologic preparation	7	41.18
**What are the obstacles to the disclosure process?**		
Parents’ opposition	15	88.23
Failure to attend the appointments	12	70.59
Insufficient number of psychologists	8	47.06
**What solutions would you suggest to improve the disclosure process?**		
Recruit psychologists	14	82.35
Improve disclosers’ training	13	76.47
Create disclosure clubs	10	58.82
Convince parents to accept HIV status disclosure	8	47.06

The square brackets show the minimum and maximum ages

### Adherence to the antiretroviral treatment

**Figure 2:**
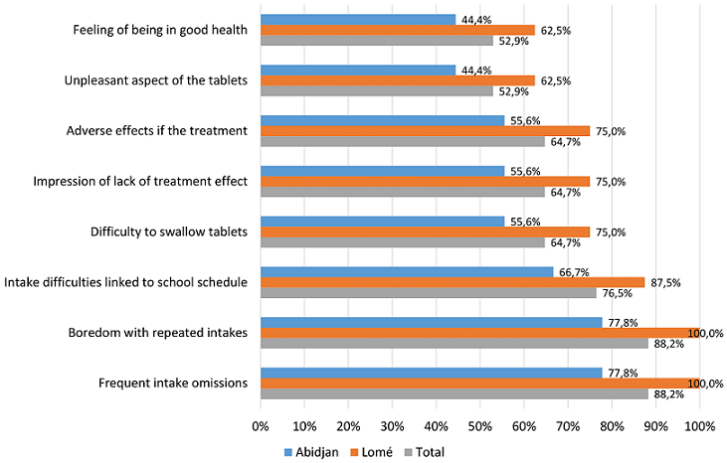
children’s and adolescents’ problems with adherence to HIV antiviral treatment

Regarding the satisfaction of the respondents with the children´s and adolescents´ adherence to HIV antiviral treatment, 11 (64.7%) were rather satisfied (of whom 2 very satisfied) and 6 (35.3%) rather unsatisfied (of whom 1 not at all satisfied). The major reasons for poor adherence cited were: boredom with repeated intakes, frequent omissions, school time constraints, difficulties to swallow the tablets, treatment adverse effects, and impression of lack of treatment effect (Figure 2). Nevertheless, 94.1% of the respondents confirmed the presence, within their departments, of support activities directed towards adolescent patients (support group, individual interviews with a psychologist, adherence clubs, home visits, and therapeutic education) (Table 1). To help improving adherence, the respondents proposed the following measures: increase the number of support group and/or adherence club sessions, sustain reminder phone calls and therapeutic education, increase the number of home visits, support therapeutic mentoring of each adolescent patient to ensure the best possible follow-up throughout his/her illness (Table 1).

## Discussion

In West Africa, studies in children and adolescents on HIV status disclosure and adherence to ART are rather limited. The present study is the first of its kind within sub-cohort COHADO. It was designed to understand first the difficulties health personnel and parents face in disclosing HIV status and supporting adherence to ART of minor patients then to identify and take measures to improve management of pediatric HIV/AIDS in IeDEA centers in West Africa. The disclosure of HIV status to infected minors is crucial for care because a better understanding of their disease and its difficult management is likely to promote their involvement and responsibility in treatment decisions [7]. In 2011, the WHO Guideline on HIV Disclosure Counselling for Children Up to 12 Years of Age recommended that “Children of school age (i.e., with cognitive skills and emotional maturity of a normally developing child of 6-12 years) should be told their HIV positive status; younger children should be told their status incrementally to accommodate their cognitive skills and emotional maturity, in preparation for full disclosure.” [8]. The results reported here are in keeping with this “Key Recommendation” (the ages at start of disclosure processes were not less than 8 years). Nevertheless, “children who are informed of their HIV status cope with disclosure as effectively, if not better, than adults” [7]. In fact, experiences stemming from counselling children with other affections than AIDS have shown that children overcome better the disclosure of the disease when they are young. It has been also proven, especially in developed countries, that children informed early about their adoption had less psychosocial problems than those informed later and who grow believing that their adoptive parents were their biological ones [7].

The age at which the disclosure should be made is still a matter of debate. Some authors advocate early disclosure (age 5 to 7 years) and believe “the sooner is the better” because adolescents are less able to overcome subsequent psychological and social complications. In fact, the process of HIV disclosure to children and adolescents is certainly a complex task and depends on several factors, but mainly on the “children's emotional and aptitudinal ability to understand and cope with the nature of the illness, stigma, family relations and concerns about social support.” [8] Thus, given the above-exposed recommendations and the study results from opinions of actors on the field (physicians, psychologists, etc.), we believe disclosure should be discussed on a case-by-case basis and be a progressive process and not a one-time event. The process start may be prompted by the child´s questions. Thus, parents or guardians should be attentive to child´s questions or comments that show his/her readiness to hear the answers.

In this study, the main obstacles to the disclosure of the HIV status to the adolescents were parents´ or guardians´ disapproval (88.2%) and the failure to attend scheduled visits (70.6%). The attempt to investigate parents´ reasons for disapproval revealed that parents or guardians feared of being blamed or even hated by their children. In a Thai study and a Kenyan study (carried out in 2012 and 2013, respectively), the main reasons were the fear from sending the child into sadness (47%) [9] or lead him to depression (24%) [10]. The obstacles to disclosure depend then on the family environment and the cultural context of the disease. Caregivers would then better insist on the benefits of a well-prepared disclosure; i.e., involvement, responsibility, and, especially, adherence to ART and retention in care [11-15]. In 2015, a South African study demonstrated that children aware of their HIV status are twice more likely to show a high adherence to the treatment [16]. In addition, the disclosure is important to decrease the probabilities of reinfection and high-risk behavior. A literature review published in 2016 has revealed that, in sub-Saharan Africa: i) the overall rate of HIV status disclosure to children and adolescents was rather low; ii) the rates of “full disclosure” and “partial disclosure” did not exceed 41% and 38%, respectively; iii) half the studies reviewed reported “no disclosure” to over 50% of children; iv) the rate of “deflected information” went up to 49.5%; and, v) the age at disclosure was quite variable across studies [17]. In fact, parents faced with the painful and difficult disclosure task may feel disoriented and helpless, which leads to repeated delays of the disclosure process.

Caregivers play essential roles in young patients´ adherence and follow-up visits. In a review meeting at Abidjan (ICASA 2017), one pediatrician approved by a psychologist said: “The problems of adherence to HIV treatment in adolescents are inherent to adolescence itself”. This view is recognized by most communities and cultures worldwide who consider adolescence as a crucial step in life marked by traditional rites of passage during which the adolescent acquires the knowledge and competence necessary for an independent life [18]. This values the roles of the disclosing psychologists who have to deal jointly with two complex contexts: AIDS and adolescence. A relatively recent study conducted in Ethiopia on caregiver-reported adherence to ART has shown that achieving optimal viral suppression in children or adolescents requires excellent adherence (intake of >95% of prescribed doses), that the observed adherence was circa 93%, and that most caregivers were satisfied [19]. In that study, one major promoter of adherence was the use of medication reminders: without reminders, the children were two to twelve times less “likely to miss the prescribed dose”. Here, the analysis of caregivers´ satisfaction with adolescents´ adherence showed that only 64.7% of them were satisfied despite the declaration by 94% of them that adherence-promoting activities do exist within their departments. Nevertheless, the results from Lomé and Abidjan indicate that promoting adherence still requires more support groups and adherence club sessions, more therapeutic education, more home visits, more therapeutic mentoring, and maintenance of medication reminder phone calls.

The present study has several strengths: i) the qualitative participatory action research approach is a known and reliable method based on triad “need-demand-response” and used by experts in the field for decision-making or corrective initiatives in health; it is further recommended by *Institut Renaudot*; ii) the participation of all solicited health professionals who showed interest and involvement in adolescents´ health, issues, and rights; iii) a relatively limited social desirability bias because the respondents were told there are no good or bad answers; iv) a relatively limited interviewer bias because all interviewers were well trained in interviewing skills and aware of research ethics and because the questionnaire was pre-tested and adjusted before use. Nevertheless, the major limitation is the small number of participants. In fact, the investigators targeted the contribution of experienced caregivers working in specialized departments likely to receive high numbers of patients and implement up-to-date HIV-related recommendations. Overall, we believe the present results provide an adequate picture of the current practices regarding HIV status disclosure and adherence problems in urban pediatric departments, at least in French-speaking countries.

## Conclusion

The disclosure of HIV status to a child or an adolescent is a very sensitive task. Its negative consequences are difficult to predict, prevent, bear, and overcome in most sub-Saharan African communities. Nevertheless, providing they are well-prepared, disclosures should be made to improve adherence to ART and retention in care. The present study was probably the first to be carried out in Togo and Ivory Coast since the creation of West Africa IeDEA. Despite its modest size and limitations, it provided interesting indications on measures to improve disclosure of HIV status and adherence to ART in children and adolescents. Nevertheless, further rigorous and larger analytical studies are still needed to better identify and quantify the factors that delay divulgation of HIV status or interfere with adherence to ART in adolescents. These studies will help designing better solutions for low-income countries, solutions that prevent ART failures and transitions to second-line ART.

### What is known about this topic

- *In Africa, the proportion of minors with AIDS is ever increasing, whereas responsible adherence to ART is essential to live with HIV; this raises problems of status disclosure and adherence with intricate individual, social, cultural, medical, and psychological factors;*
- *Despite specific campaigns and implementation of international recommendations, the results of disclosure processes and adherence-improving measures are still suboptimal in several African countries*.

### What this study adds

- *Questionnaires to health professionals and parents´ interviews investigated the conditions of disclosure and adherence in two major hospitals in Lomé (Togo) and Abidjan (Ivory Coast) as a reflection of African urban conditions;*
- *Internationally recommended measures to improve disclosure processes and adherence to treatments are taken in these urban hospitals but still face mainly cultural, staffing, practical, and organizational difficulties;*
- *Even in large urban facilities where disclosure- and adherence-improving measures are taken, more efforts have to be made toward increasing patients´ awareness, increasing the number of specialized personnel, raising personnel´s skills and motivation, and promoting local activities (calls, home visits, support groups, patients´ clubs, therapeutic mentoring, etc.)*.
